# Editorial: Insights in Alzheimer's disease and related dementias

**DOI:** 10.3389/fnagi.2022.1068156

**Published:** 2022-11-23

**Authors:** Agustín Ibáñez, Allison B. Reiss, Nilton Custodio, Suvarna Alladi

**Affiliations:** ^1^Latin American Brain Health Institute (BrainLat), Universidad Adolfo Ibáñez, Santiago, Chile; ^2^Cognitive Neuroscience Center (CNC), Universidad de San Andrés and CONICET, Buenos Aires, Argentina; ^3^Global Brain Health Institute (GBHI), University of California, San Francisco (UCSF), San Francisco, CA, United States; ^4^Trinity College Dublin (TCD), Dublin, Ireland; ^5^Department of Medicine and Biomedical Research Institute, NYU Long Island School of Medicine, Mineola, NY, United States; ^6^Department of Neurology, Instituto Peruano de Neurociencias, Lima, Peru; ^7^Unit of Diagnosis of Cognitive Impairment and Dementia Prevention, Instituto Peruano de Neurociencias, Lima, Peru; ^8^Department of Neurology, National Institute of Mental Health and Neurosciences, Bengaluru, India

**Keywords:** dementia, Alzheimer's disease (AD), ADRD, research, clinical, imaging, genetics

## Introduction

According to the World Alzheimer's Report (Alzheimer's Disease International, 2022) and the Global status report on the public health response to dementia (WHO, 2022), 55+ million people live with dementia, a condition characterized by deterioration in cognition and functionality beyond the expected effects of normal aging. WHO has recognized dementia as a public health priority. Alzheimer's disease and related dementias (ADRD) may result from a disparate combination of processes, including genetic and environmental factors (fiscal, social, economic), pathological processes, and injuries that primarily or secondarily affect the brain. Dementia is a leading cause of disability, dependence and death among older people globally. The impact of dementia at personal and societal levels is unequally distributed, usually following a distribution similar to that of the inequality index of the corresponding country.

Current dementia research is transitioning from more simplistic and universal models toward complex, multilevel, heterogeneity-sensitive, and diversity-oriented frameworks. These landscapes of dementia science are changing rapidly, creating novel bridges across disciplines, diverse populations, regions, scales, methods and approaches. Some of these reconfigurations are driven by animal and human research focused in multiple emerging areas such as diversity contributions to genetic traits (Dehghani et al., 2021); heterogeneity and variation in protein misfolding and aggregation (Frisoni et al., 2022); explanatory models based on excitation/inhibition synaptic activity (Babiloni et al., 2020); impact of multiple sources of disparities (gender, admixtures, cultural, socioeconomic) (Alladi and Hachinski, 2018; Parra et al., 2018, 2021); development of multimodal and region-specific biomarkers (Moguilner et al., 2022; Parra et al., 2022; Maito et al., 2023) and initiatives (Ibanez et al., 2021; Parra et al., 2021; Duran-Aniotz et al., 2022); interactions between environmental stressors and physiopathological mechanisms of allostatic overload (Birba et al., 2022; De Felice et al., 2022; Migeot et al., 2022); and going beyond universal models toward non-stereotypical samples (Greene et al., 2022) and designs (Ibanez, 2022) in neuroscience and dementia (Alladi and Hachinski, 2018). Notably, many of these key matters are being covered in this special issue.

## The contributions to ADRD

This Research Topic comprises 20 articles, involving >150 authors across the globe, and organized into four main areas: (a) animal studies, and human studies of (b) biomarkers and basic approaches, (c) neurocognition, and (d) clinically relevant assessments. These contributions are briefly summarized in the following sections.

### Animal studies

Moreno-Gonzalez et al. were interested in testing the hypothesis that protein misfolding and aggregation can be induced by administration of small quantities of preformed aggregates. They model their hypothesis on a principle similar to that of prion diseases, which are transmitted by a proteinaceous infectious agent that seeds misfolding and aggregation of the human prion protein. Using transgenic animal models of AD, they assessed how the intra-cerebrally inoculated Aβ aggregates in the brain of aged cattle promote AD pathological features. Results suggest that aged cattle can develop AD-like neuropathological abnormalities (amyloid plaques). Also, bovine-derived aggregates accelerate Aβ amyloid deposition.

Jiang et al. compared AD postmortem brain tissue with brains from two types (AppNL–F and AppNL–G–F) of APP knock-in mice, which typically exhibit robust Aβ pathology. They found an increase of both p62 and LC3-II levels in the brains of the knock-in mice compared to wild type mice, signaling inhibited autophagy, and discovered LC3-positive puncta in the hippocampus of AppNL–F mice around the Aβ plaques. They posit that APP knock-in mouse models are a promising platform for aiding in the correlation of Aβ and autophagy.

Zou et al. researched amyloid plaques and neuronal loss in AD murine models. They found an increase in reactive oxygen species (ROS) in pre-senilin (PS) deficient fibroblasts, while H202 and ferrous sulfate treatments produced more ROS in PS deficient fibroblasts over wild-type fibroblasts. Their results also suggest reduced iron sequestration in PS deficient cells, as well as a key role of γ-secretase activity in maintaining ferritin levels. Overexpression of PS1 mutants in wild type fibroblasts decreased ferritin light chain levels while increasing intracellular ROS levels. Their results suggest how PS dysfunction can reduce intracellular ferritin levels, resulting in free iron-induced oxidative stress which may play an important role in AD pathogenesis.

Hurley et al. reported the whole-genome sequencing and analysis of the degu genome. The degu is a diurnal long-living rodent that can develop changes analogous to human aging and AD. Their results revealed unique features and molecular adaptations similar to aging and AD in humans. Particularly, they identified a novel APOE gene variant that correlated with an increase in the amyloid plaques of the brain. Their results could help further advance biomedical treatments for AD.

### Human studies of biomarkers and basic advances

Kim et al. looked at the issue of whether Aβ accumulation in the cortex and striatum, as well as tau accumulation, differ depending on sex and APOE genotypes. Using a sample of subjects from the Alzheimer's Disease Neuroimaging Initiative database, they selected 534 subjects who had undergone 18F-florbetapir PET and 163 subjects who had undergone 18F-flortaucipir PET. They performed trajectory analysis of Aβ and tau protein deposition and obtained predictions of SUVR curves across time. They found no differences of cortical Aβ accumulation depending on sex, but did find striatal Aβ accumulation was faster for women than for men. This difference was even more pronounced for tau accumulation. Still, APOE ε4 carriers showed greater progression than non-carriers, regardless of the biomarkers' trajectories.

Unnur et al. evaluated the association between CSF cholinergic enzymes with AD-related biomarkers and cognitive functioning. They measured enzyme activity of AChE and BuChE in the CSF, as well as amyloid-β1–42 (Aβ42), phosphorylated tau (P-tau), total-tau (T-tau), neurofilament light (NFL), YKL-40, S100 calcium-binding protein B (S100B), and glial fibrillary acidic protein (GFAP). They also evaluated verbal episodic memory with behavioral batteries. Although they did not find a relationship between CSF Aβ42 and AChE or BuChE activity, they did find a positive correlation between higher activity of ACh-degrading cholinergic enzymes and increased neurodegeneration, neurofibrillary tangles and inflammation in pre- and early dementia.

Lai et al. explored the cuproptosis-related molecular clusters in AD and also developed a predictive model with a sample of 310 AD patients from the GSE33000 microarray dataset. They found cluster-specific differentially expressed genes in 2 clusters. Cluster1 was related to synapse and axon regulation while Cluster2 was involved in immune responses. High diagnostic value subtype-specific genes were identified with a random forest machine learning model and the 5 genes found were externally validated with two datasets. All of the five model-related genes were significantly associated with Aβ-42 levels and β-secretase activity, illustrating the complex interaction of cuproptosis and AD.

The amyloid hypothesis of AD pathogenesis is still one of the main drivers of research. However, the results of the anti-Aβ antibody, aducanumab, are still uncertain. Accounting for divided opinions on Aβ as a major causal factor of AD, Kawabata et al. proposed a novel hypothesis on how excessive/aberrant and maladaptive synaptic plasticity are the basis for AD pathophysiology.

### Human studies of neurocognition in ADRD

Chen et al. assessed abnormal functional connectivity in posterior cortical atrophy (PCA) and semantic dementia (SD). They evaluated seed-based functional connectivity in PCA, SD, and control subjects, along with detailed clinical, physical and neuropsychological assessments. Their results revealed abnormal connectivity within the cortex in the language and salience networks for both PCA and SD patients. Meanwhile, functional connectivity changes in the visual networks were unique for PCA patients. All FC changes were matched for cognitive deficits and accounted for abnormal metabolism.

Tan et al. evaluated the behavioral and neural associations between olfactory identification and cognitive functioning in a sample of 645 adults (AD and MCI) from the Taizhou Imaging Study. They found that higher olfactory identification score on smell testing was associated with better scores on a battery of neuropsychological tests of cognitive function. Higher olfactory identification was correlated with lower likelihood of MCI and dementia. Amygdala volume was significantly correlated with olfactory identification and the results from the cognition batteries, thus, highlighting a key role of the amygdala in the link between olfactory identification and cognitive function.

Hoong Kang et al. evaluated the correlation between performance in language tests and cortical thickness in order to determine neural substrates of 96 Korean patients with primary progressive aphasia. Poor performance on language tests (object naming, semantic generative naming, phonemic generative naming and comprehension) was correlated to lower cortical thickness in key cortical regions. Specifically, the neural substrates of the semantic generative naming test (midportion of the lateral and basal temporal regions) significantly differed from control patients with other dementia subtypes.

Song et al. assessed alterations in resting-state functional connectivity density (FCD) in subjective cognitive decline (SCD), amnestic MCI (aMCI), and controls (*N* = 194) in order to further define how these changes can help to distinguish preclinical and early-stage AD. Their results revealed global FCD in the left parahippocampal gyrus and increased FCD in the left hippocampus for SCD patients. Meanwhile, aMCI patients exhibited decreased global and long-range FCD in the left parahippocampal gyrus. Follow-up FC analysis revealed significant variations between the left parahippocampal gyrus and occipital lobe in SCD and aMCI patients. These results can help understand the progression of SCD and aMCI to AD.

Chen et al. determined whether Sortilin-related receptor 1 (SORL1) polymorphisms were associated with volume differences in brain regions in late-onset AD in patients of Han Chinese descent. They recruited 200 late-onset AD patients (Taipei Veterans General Hospital) for MR imaging and neurocognitive assessment, with 77.5% of patients receiving follow-up Mini-Mental State Examination 2 years after enrollment to assess changes longitudinally. They found that the homozygous rs2298813 allele was associated with larger volumes in the right putamen and pallidum (which was correlated with verbal fluency). The major and minor alleles of rs2298813 predicted clinical progression in the 2-year follow-up, while putaminal volume was associated with verbal fluency.

Laczó et al. examined spatial navigation as a cognitive marker of clinical and preclinical AD. For this study, they assessed spatial navigation performance in older adults with AD aMCI (positive amyloid PET and/or CSF AD biomarkers) vs. non-AD aMCI (negative amyloid PET and/or normal CSF amyloid-β1–42), and assessed navigation performance and MRI measures of regional brain volumetry. Their results revealed that AD aMCI adults performed worse than non-AD aMCI adults in route learning. Meanwhile, spatial navigation impairments were associated with posterior medial temporal lobe and parietal atrophy and reflected AD pathology.

Oh et al. looked for the pathological and functional correlates of extraversion and neuroticism in healthy adults and older participants in order to gain better understanding of how personality traits can be crucial for vulnerability to or protection from AD. They measured brain imaging *via* a task-switching fMRI paradigm in young adults, while they obtained data on Aβ deposition in older individuals *via* PET. They found that extraversion was significantly associated with lower Aβ across brain regions in older individuals, while high extraversion in young adults was associated with lower activity in the anterior cingulate cortex, left anterior insular cortex, left putamen, and medial frontal gyrus. Higher neuroticism was overall associated with increased global brain activity. Results suggest that extraversion, *via* efficient neuronal activity, may confer protection against Aβ pathology.

### Human studies assessing ADRD clinically relevant measures

Zhang et al. studied the top 100 published papers on AD and epilepsy. They found a substantial increase in publications between the years 2000 to 2021, with a mean of 67.4 citations for these 100 papers. The US, and Columbia University were the most influential country and institution, respectively, while Journal of Alzheimer's Disease accounted for the highest number of papers in this area (*n* = 8). Their results point toward the increasing importance of this topic and the need for fruitful collaborations and cooperation going forward.

Kang et al. aimed to determine the association of BMI changes and variability with Aβ positivity. They conducted a large retrospective cohort study with participants 50 years of age and above using multivariable logistic regression. They found that decreased or increased BMI, as well as BMI variability, were positively correlated with greater Aβ positivity. Their results showcase how BMI changes, and particularly BMI variability, make the brain more prone to Aβ deposition, as well as signaling important interventions in preventing Aβ deposition with weight control and stabilization.

Chang et al. looked at the relationship of coenzyme Q10 with dementia biomarkers and antioxidant capacity in 80 dementia patients. They found a majority of patients (73%) presented with low coenzyme Q10 levels. Coenzyme Q10 was inversely correlated with plasma amyloid β-42 and amyloid β-42/40 ratio, but not with tau level. Coenzyme Q10 levels correlated positively with antioxidant capacity. Their results show that it may be beneficial to monitor and maintain adequate levels of coenzyme Q10 in patients with dementia.

Finally, Wang et al. examined the effects on cognitive function of serum uric acid (SUA) at baseline and with change after 4 years in a non-normotensive population from the China Health and Retirement Longitudinal Study (CHARLS; *N* = 3,905). They highlighted four single-trajectories of global cognitive performance, executive function and episodic memory. Overall, higher SUA levels were associated with favorable cognitive trajectories and a moderate increase of SUA over time was good for cognitive function as long as SUA was in the normal range. However, persistent hyperurcemia resulted in increased risk of cognitive dysfunction. This indicates the importance of maintaining normal SUA levels.

## Conclusions

We are now entering the third decade of the 21st century, and, especially in the last years, the achievements made by scientists have been exceptional, leading to major advancements in the fast-growing field of ADRD. The present Research Topic highlights some of the latest advancements, new insights, novel developments, current challenges, latest discoveries, and future perspectives in ADRD research. We hope these contributions may shed light on the progress made in the past and in the forthcoming challenges of the field. We are proud of the diversity of our authors, topics, and editorial team and hope they will contribute to a more diverse and robust science of dementia.

## Author contributions

AI prepared the initial draft of this editorial. AR, SA, and NC carefully revised the draft. All authors contributed to the contents of this article and approved the final version.

## Funding

AI is partially supported by grants from CONICET; ANID/FONDECYT Regular (1210195, 1210176, and 1220995); ANID/FONDAP/15150012; ANID/PIA/ANILLOS ACT210096; FONDEF ID20I10152, ID22I10029; ANID/FONDAP 15150012; Takeda CW2680521 and the MULTI-PARTNER CONSORTIUM TO EXPAND DEMENTIA RESEARCH IN LATIN AMERICA [ReDLat, supported by National Institutes of Health, National Institutes of Aging (R01 AG057234), Alzheimer's Association (SG-20-725707), Rainwater Charitable foundation – Tau Consortium, and Global Brain Health Institute]. AR is supported by the Alzheimer's Foundation of America (Award AWD00004772) and The Herb and Evelyn Abrams Family Amyloid Research Fund. The contents of this publication are solely the responsibility of the authors and do not represent the official views of these Institutions.

## Conflict of interest

The authors declare that the research was conducted in the absence of any commercial or financial relationships that could be construed as a potential conflict of interest.

## Publisher's note

All claims expressed in this article are solely those of the authors and do not necessarily represent those of their affiliated organizations, or those of the publisher, the editors and the reviewers. Any product that may be evaluated in this article, or claim that may be made by its manufacturer, is not guaranteed or endorsed by the publisher.
